# Molecular characterization of closteroviruses infecting *Cordyline fruticosa* L. in Hawaii

**DOI:** 10.3389/fmicb.2013.00039

**Published:** 2013-03-05

**Authors:** Michael J. Melzer, Jari S. Sugano, Janice Y. Uchida, Wayne B. Borth, Michael K. Kawate, John S. Hu

**Affiliations:** Department of Plant and Environmental Protection Sciences, College of Tropical Agriculture and Human Resources, University of HawaiiHonolulu, HI, USA

**Keywords:** pyrosequencing, *Closteroviridae*, *Velarivirus*,* Cordyline*, ti ringspot

## Abstract

In Hawaii, common green ti plants (*Cordyline fruticosa* L.) have been shown to harbor Cordyline virus 1 (CoV-1) which, along with Little cherry virus 1 (LChV-1), and Grapevine leafroll-associated virus 7 (GLRaV-7), form a distinct clade within the family *Closteroviridae*. Preliminary work has indicated that, aside from CoV-1, three additional closteroviruses may infect common green ti plants in Hawaii. In this study, pyrosequencing was used to characterize the genomes of closteroviruses infecting a single common green ti plant. The sequence data confirmed the presence of CoV-1 as well as three additional closteroviruses. Although all four viruses had the same general genome organization, the sequence divergence between the RNA-dependent RNA polymerase, heat shock protein 70 homolog, and coat protein ranged from 22 to 61%, indicating these represent four distinct closterovirus species. The names CoV-2, CoV-3, and CoV-4 are proposed for the three new viruses. Phylogenetic analyses placed CoV-2, CoV-3, and CoV-4 in the same clade as CoV-1, LChV-1, and GLRaV-7.

## INTRODUCTION

The family *Closteroviridae* represents a related group of mono- and multipartite, single-stranded, positive-sense RNA plant viruses with long, flexuous virions (Dolja et al., 1994). There are currently three genera in the family which are segregated largely based on vector species. Members of the genera *Closterovirus*, *Crinivirus*, and *Ampelovirus* are, in general, transmitted by aphids, whiteflies, and mealybugs, respectively (Karasev, 2000). Members of the genera *Closterovirus* and *Ampelovirus* have monopartite genomes, while members of the genus *Crinivirus* have multipartite genomes. Little cherry virus 1 (LChV-1) and Grapevine leafroll-associated virus 7 (GLRaV-7) are two members of the family that have not been assigned to a genus (Martelli et al., 2002). Molecular phylogenies and sequence similarity values associate them most closely to members of the genus *Crinivirus*, however, their monopartite genomes and their lack of a known insect vector have precluded their inclusion into this genus.

The ti plant, *Cordyline fruticosa* (L.), belongs to the plant family Agavaceae and has considerable cultural and economic importance in Hawaii and throughout most of Polynesia. In Hawaii, the common green variety was introduced by early Polynesian settlers and is a popular ornamental in residential settings that has also become naturalized in Hawaii’s forests. Vegetatively propagated due to sterility (Hinkle, 2007), it is also the most prominent variety grown commercially. In 2009, ti farmers on the island of Oahu reported ringspot symptoms on their common green ti plants. These ringspot symptoms were subsequently observed on commercial and residential ti plants on the islands of Maui and Hawaii. In a search for a causal agent of the ringspot symptoms, it was recently discovered that Hawaiian ti plants harbored multiple putative members of the plant virus family *Closteroviridae* (Melzer et al., 2011). The complete nucleotide sequence for one of these viruses, Cordyline virus 1 (CoV-1), was determined. Its 16.9 kb genome was organized similar to LChV-1 and GLRaV-7 (Melzer et al., 2011). Together, LChV-1, GLRaV-7, and CoV-1 form a monophyletic clade distinct from the other three genera in the family *Closteroviridae*. This has led to proposals for the creation of a fourth genus, “*Velarivirus*,” to represent these viruses (Al Rwahnih et al., 2012; Martelli et al., 2012).

A reverse-transcription polymerase chain reaction (PCR assay revealed that CoV-1 is widespread in Hawaii and is present in ti plants with and without ringspot symptoms, making it unlikely to be involved in the etiology of the disease (Melzer et al., 2011). Based on partial sequence data, three additional closteroviruses were identified in ti plants. The objectives of this study are to further characterize these additional closteroviruses in common green ti plants and determine whether they represent distinct species (or strains of CoV-1) as well as their placement within the family *Closteroviridae*.

## MATERIALS AND METHODS

### LIBRARY SEQUENCING AND ASSEMBLY

A previously described randomly primed complementary DNA (cDNA) library generated from double-stranded (ds) RNA isolated from a common green ti plant was used as the input material for multiplex pyrosequencing (Melzer et al., 2011). The most common cDNA length was estimated to be approximated 550 bp based on agarose gel electrophoresis. MID7 (5′-ACGTACACACT-3′) was ligated to the cDNAs which then underwent pyrosequencing using a 454 GS FLX Titanium platform (Roche, Branford, CT, USA) at the University of Hawaii’s Advanced Studies in Genomics, Proteomics and Bioinformatics (ASGPB) laboratory. Following pyrosequencing, the MID7 and random primer sequences as well as low quality basecalls at the end of reads were trimmed. Short length (<65 nt) and low quality reads as well as those that mapped to the CoV-1 genome were purged from the dataset. The remaining reads underwent *de novo* assembly using Geneious® Pro 5.6.5 (Biomatters Ltd., Auckland, New Zealand). To validate low coverage regions or where unexpected stop codons or frameshifts occurred, and to bridge sequence gaps between contiguous sequences (contigs), primers were designed flanking the region in question and PCR was performed using the cDNA library as template. PCR products were either directly sequenced following treatment with ExoSAP-IT® (USB/Affymetrix, Santa Clara, CA, USA) or ligated into pGEM®-T Easy (Promega, Madison, WI, USA) followed by Sanger-based sequencing at the ASGPB.

The 3′-terminal sequences were obtained by polyadenylating heat-denatured dsRNA using yeast poly(A) polymerase (USB/Affymetrix) following the manufacturer’s instructions. An oligo d(T) primer [5′-CACTCCCTATTATCCAGG(T)_16_-3′] was used to initiate cDNA synthesis and also used in the subsequent PCR reaction along with a virus-specific primer designed to anneal near the 3′-end of the available virus sequence. Amplification products were cloned using pGEM®-T Easy and at least five clones underwent Sanger-based sequencing at the ASGPB.

### PHYLOGENETIC ANALYSES

The combined helicase domain (HEL), RNA-dependent RNA polymerase (RdRp), heat shock protein 70 homolog (HSP70h), p61 (PF03225), and coat protein (CP) amino acid sequences of members and tentative members of the family *Closteroviridae* were aligned using ClustalX 2.0.12 (Larkin et al., 2007). With this alignment, the phylogenetic relationships of the sequences were inferred using neighbor-joining (NJ) and maximum likelihood (ML) algorithms. The NJ algorithm was performed using ClustalX 2.0.12 and bootstrapped with 1000 replications. The ML algorithm was performed with PhyML 3.0 (Guindon et al., 2010) using the WAG model and bootstrapped with 1000 replications.

## RESULTS

### LIBRARY SEQUENCING AND ASSEMBLY

A total of 107,655 high quality reads >64 nt were generated from the cDNA library, with maximum, minimum, and mean lengths of 772, 65, and 392.1 nt, respectively. Of these reads, 4,424 mapped to the CoV-1 genome. The majority of the remaining reads assembled into three contigs, each in excess of 10 kb in length (**Table 1**). Based on comparisons with accessions in GenBank, all three contigs represented closterovirus-based genomes. The first contig was found to be essentially identical to the previously identified Contig5 (Melzer et al., 2011) where the two sequences overlapped, and was thus designated Contig5. Similarly, the second and third contigs were found to be essentially identical in overlapping regions with Contig8 and CloneH11, respectively, and were designated as such. The 15,031 nt Contig5 was extended to 15,107 nt following the addition of the 3′-terminal sequence. The 14,941 nt Contig8 was extended to 16,274 nt following the addition of the 3′-terminal sequence and a contig in the 5′-region of the genome. The 10,684 nt CloneH11 was extended to 14,620 nt with the addition of the 3′-terminal sequence and a contig in the 5′-region of the genome. The 5′-terminal sequences were not obtained for any of the contigs.

**Table 1:** Summary of pyrosequencing results and assembly of reads into contiguous sequences (contigs).

	# of reads (% of total)	Length of contig^1^	Mean coverage
CoV-1	4,424 (4.1)	n/a	n/a
CoV-2 (Contig5)	11,395 (10.6)	15,031	342.8
CoV-3 (Contig8)	79,593 (73.9)	14,941	2410.8
CoV-4 (CloneH11)	3,537 (3.3)	10,684	168.7
Unincorporated	8,728 (8.1)	n/a	n/a

1 *Post-editing*.

### GENOME ORGANIZATION

**FIGURE 1:**
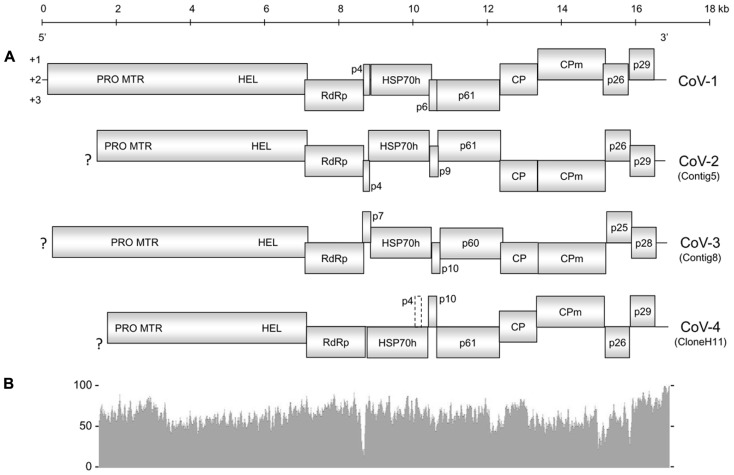
**(A)** Genome organization of Cordyline virus 1 (CoV-1), CoV-2, CoV-3, and CoV-4. Solid boxes represent putative open reading frames (ORFs) with their vertical position indicating reading frame. The dashed box in CoV-4 represents a possible ORF that encodes a small hydrophobic protein within the HSP70h ORF. PRO, protease domain; MTR, methyltransferase domain; HEL, helicase domain; RdRp, RNA-dependent RNA polymerase; HSP70h, heat shock protein 70 homolog; CP, coat protein; CPm, minor coat protein; ?, incomplete terminus. **(B)** Graphical comparison of the percent nucleotide identity between CoV-1 and CoV-2 where the two genomes overlap. The above scale gives approximate size in kilobases for both **(A)** and **(B)**.

The overall genome organization of Contig5, Contig8, and CloneH11 were similar to that of CoV-1 (**Figure 1**), GLRaV-7, and LChV-1 (Jelkmann et al., 1997, 2012; Melzer et al., 2011). Although incomplete at their 5′-terminal regions, open reading frame (ORF)1a of Contig5, Contig8, and CloneH11 encoded a polyprotein containing protease (PRO; PF05533), methyltransferase (MTR; PF01160), and HEL (PF01443) domains. ORF1a of Contig5 and Contig8 terminated with the sequence 5′-UUUGA-3′ with the stop codon underlined. This is also the terminal sequence of CoV-1 and GLRaV-7 (Melzer et al., 2011; Jelkmann et al., 2012) and may initiate a +1 ribosomal frameshift allowing expression of ORF1b. ORF1a of CloneH11 terminated with the sequence 5′-UUUAA-3′ that may also allow expression of ORF1b via the same frameshift mechanism. For all contigs, however, a start codon was present near the ORF1a termination sequence in the same reading frame of ORF1b that may also allow its expression. This was also observed for CoV-1 and LChV-1 (Jelkmann et al., 1997; Melzer et al., 2011). ORF1b of all three contigs encoded all the typical motifs of an RdRp (Koonin, 1991). Small transmembrane proteins 4 and 7 kDa in weight were present between ORF1b and the HSP70h ORF for Contig5 and Contig8, respectively. No such protein was present at this location in CloneH11, however, a 4 kDa protein with transmembrane properties was present in the +1 reading frame within C-terminal region of the HSP70h ORF (which is in the +3 reading frame). For all contigs, downstream of the HSP70h ORF was a 9–10 kDa ORF that is also present in CoV-1 and GLRaV-7, followed by ORFs encoding a 60–61 kDa protein common to all closteroviruses (PF03225), the major CP (PF01785), and then the minor CP (CPm). An ORF encoding a 25–26 kDa homolog of CoV-1 p26 was present in Contig5, Contig8, and CloneH11. The final ORFs encoded by Contig5, Contig8, and CloneH11 were 28–29 kDa proteins. While p29 of Contig5 and p28 of Contig8 shared sequence homology with p29 of CoV-1, p29 of CloneH11 did not appear to be a homolog of these putative proteins, and did not have significant sequence similarity with any viral sequences currently in GenBank. The 3′-untranslated regions (UTRs) of Contig5, Contig8, and CloneH11 were 259, 154, and 186 nt, respectively. The exact 3′-termini of Contig5 and CloneH11 were identical to that of CoV-1, having the sequence 5′...AAAGGUGCG-3′. Contig8 also ended with this sequence, but appeared to lack the terminal guanine residue.

### DIVERSITY AND PHYLOGENETIC PLACEMENT

The amino acid identity between Contig5, Contig8, CloneH11, and CoV-1 for their respective RdRp, HSP70h, and CP sequences was under 70% in all cases except for the RdRp sequences of Contig5 and CoV-1 which were 78% identical (**Table 2**). Using the current criteria for closterovirus species demarcation recently revised by the International Committee on Taxonomy of Viruses (Martelli et al., 2011), these contigs would represent distinct closterovirus species. As such, Contig5, Contig8, and CloneH11 were tentatively designated CoV-2, CoV-3, and CoV-4, respectively.

**Table 2:** RNA-dependent RNA polymerase/heat shock protein 70 homolog/coat protein amino acid percent identities between the CoVs infecting common green ti plants.

	CoV-1	CoV-2 (Contig5)	CoV-3 (Contig8)
CoV-2 (Contig5)	78/69/67		
CoV-3 (Contig8)	62/50/44	62/51/41	
CoV-4 (CloneH11)	59/53/39	57/51/40	55/55/39

Cordyline virus 1 and CoV-2 appear to be the most closely related of the CoVs characterized in this study with an overall nucleotide identity of 63.7%. Toward the 3′-termini of their genomes, however, the similarity gradually increased. This similarity peaked in the 3′UTR of CoV-1 and CoV-2 which shared a 90.8% nucleotide identity.

Over 8% of the total sequence reads did not map to the genomes of CoV-1, CoV-2, CoV-3, or CoV-4 (**Table 1**). The majority of these appeared to be either of plant or prokaryotic origin, or did not share significant similarity to any of the sequence accessions in the current databases. Approximately 31% of these reads, however, represented closterovirus sequences. One thousand and ninety-five of these reads were selected for further investigation. One hundred and seventy-four of these reads, when translated to amino acid sequences, were similar to the N-terminal region of a closterovirus ORF1a. Reverse-transcription PCR revealed this region was part of CoV-4. The remaining reads, when translated to amino acid sequence, had high similarity (between 80 and 92% identity) to proteins encoded by CoV-1.

**FIGURE 2:**
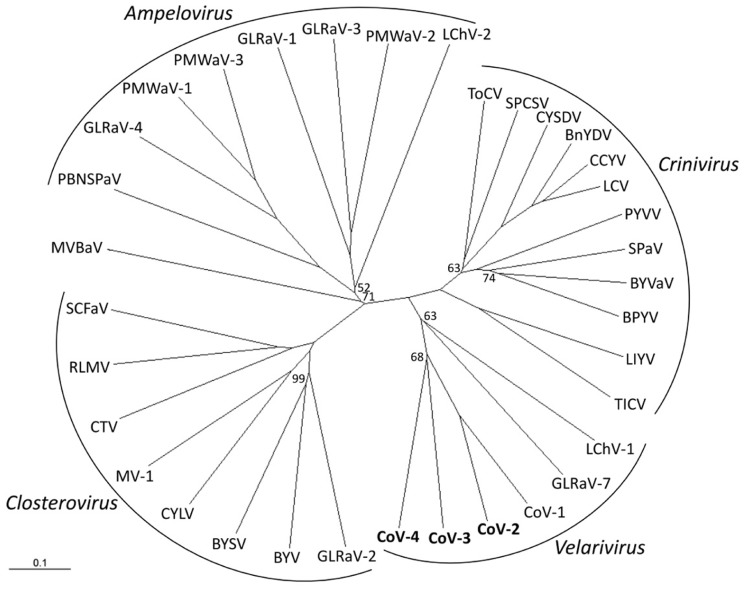
**Phylogenetic placement of Cordyline virus 2 (CoV-2), CoV-3, and CoV-4 within the family *Closteroviridae* using the combined RNA-dependent RNA polymerase, heat shock protein 70 homolog, p60, and coat protein amino acid sequences with a neighbor-joining algorithm and 1000 bootstrap replications**. Branch support is indicated in percentage support, and is 100% unless indicated, and the scale provides branch distance for the given number of substitutions. Virus abbreviations (GenBank accession numbers) are: Genus *Ampelovirus*: GLRaV-1, *Grapevine leafroll associated virus 1* (AF195822); GLRaV-3 (NC_004667); GLRaV-4 (NC_016416); LChV-2 (AF416335); PBNSPaV, *Plum bark necrosis stem pitting associated virus* (YP_001552326); PMWaV-1, *Pineapple mealybug wilt associated virus 1* (AF414119); PMWaV-2 (AF283103); PMWaV-3 (DQ399259); Genus *Closterovirus*: BYSV, *Beet yellow stunt virus* (U51931); BYV, *Beet yellows virus* (AF190581); CTV, *Citrus tristeza virus* (NC_001661); CYLV, *Carrot yellow leaf virus* (NC_013007); GLRaV-2 (AF039204); MV-1, *Mint virus-1* (NC_006944); RLMV, Raspberry leaf mottle virus (NC_008585); SCFaV, Strawberry chlorotic fleck-associated virus (DQ860839); Genus *Crinivirus*: BnYDV, *Bean yellow disorder virus* (NC_010560/NC_010561); BPYV, *Beet pseudoyellows virus* (NC_005209/NC_005210); BYVaV, *Blackberry yellow vein-associated virus* (NC_006962/NC_006963); CCYV, Cucurbit chlorotic yellows virus (AB523788/AB523789); CYSDV, *Cucurbit yellow stunting disorder virus* (NC_004809/NC_004810); LCV, *Lettuce chlorosis virus* (NC_012909/NC_012910); LIYV, *Lettuce infectious yellows virus* (NC_003617/NC_003618); PYVV, *Potato yellow vein virus* (NC_006062/NC_006063), SPaV, *Strawberry pallidosis-associated virus* (NC_005895/NC_005896); SPCSV, *Sweet potato chlorotic stunt virus* (NC_004123/NC_004124); TICV, *Tomato infectious chlorosis* virus (NC_013258/NC_013259); ToCV, *Tomato chlorosis virus* (NC_007340/NC_007341); Genus “*Velarivirus*” (proposed): CoV-1 (HM588723); CoV-2 (JQ599282); CoV-3 (JQ59983); CoV-4 (JQ599284); LChV-1, Little cherry virus 1 (NC_001836); GLRaV-7 (NC_016436); unassigned: MVBaV, Mint vein banding associated virus (AY548173).

Phylogenetic analyses of the combined HEL domain, RdRp, HSP70h, p60/61, and CP amino acid sequences using distance-based (NJ) and character-based (ML) algorithms inferred almost identical relationships between CoV-2, CoV-3, and CoV-4 and members of the family *Closteroviridae*. Both analyses clearly placed these four viruses along with CoV-1, GLRaV-7, and LChV-1 in a distinct clade within the family (**Figure 2**; data not shown).

## DISCUSSION

We have previously reported the presence of one closterovirus, CoV-1, infecting common green ti plants in Hawaii, and provided preliminary evidence for the presence of additional closterovirus species using a Sanger-based sequencing approach (Melzer et al., 2011). In this study we used a massively parallel sequencing approach to identify, in addition to CoV-1, three new closterovirus species which we have tentatively designated CoV-2, CoV-3, and CoV-4.

Cordyline virus 2 and CoV-3 share the same general genome organization as CoV-1, and differ from each other only in the molecular weight of their small hydrophobic proteins and the small protein encoded by the ORF located between their HSP70h and p61 ORFs. CoV-4, however, is unusual by lacking an ORF encoding a small transmembrane protein between the ORFs of the RdRp and HSP70h. The small transmembrane protein of *Beet yellows virus* (BYV) associates with the host endoplasmic reticulum and is involved in the cell-to-cell movement of BYV and presumably other closteroviruses as well (Peremyslov et al., 2004). An ORF which could encode a small protein possessing a transmembrane domain does exist in CoV-4, although it is located within the HSP70h ORF in a +1 reading frame relative to the HSP70h ORF. If this ORF is not expressed, it is possible that CoV-4 requires co-infection with another closterovirus for cell-to-cell movement. Since all four CoVs were present in a single host plant, multiple infections in a single host plant may not be uncommon.

Overall, the genomes of CoV-1 and CoV-2 had a moderate sequence similarity. Near the 3′-terminus, however, this similarity gradually increased to the point where the 3′UTRs of these viruses were nearly identical, indicative of a potential recombination event. Putative examples of closterovirus recombination are abundant (Cuellar et al., 2008; Melzer et al., 2010; Farooq et al., 2013). The gradual increase in sequence similarity is comparable to that proposed for Citrus tristeza virus (CTV) strain T36, and suggests the potential recombination event was not recent (Mawassi et al., 1996). The presence of multiple CoVs in a single host plant would provide an environment conducive for such recombination events.

The family *Closteroviridae* Subcommittee to the International Committee on Taxonomy of Viruses (ICTV) has recently amended a commonly used criterion for species demarcation of closteroviruses. To be considered a distinct species, the level of sequence divergence in a phylogenetically informative protein (RdRp, HSP70h, or CP) was raised from 10 to 25% (Martelli et al., 2011). This increase in stringency was undertaken to address the proliferation of closteroviruses that had a similar genome organization, host range, and biological properties but, in some cases, only marginally exceeded the previous 10% sequence divergence criterion, thereby elevating them to species status (Martelli et al., 2012). Following the implementation of this more stringent criterion, a group of seven GLRaVs species and their “variants” (GLRaV-4, GLRaV-5, GLRaV-6, GLRaV-6 DE, GLRaV-9, GLRaV-Car, and GLRaV-Pr) were condensed into a single species, GLRaV-4 (Martelli et al., 2012). Based on the amino acid identities of the RdRp, HSP70h, and CP sequences it is clear that CoV-1, CoV-3, and CoV-4 are distinct species under the new criterion. The two most closely related CoVs, CoV-1 and CoV-2, have amino acid divergence values for the RdRp, HSP70h, and CP of 22, 31, and 33%, respectively. Although the sequence divergence between the CoV-1 and CoV-2 RdRp does not exceed the 25% threshold, the average sequence divergence for these three phylogenetically informative proteins is 29%, and we therefore contend that CoV-1 and CoV-2 should represent two distinct species. Additional closterovirus-like sequences were also identified in the library. The majority of these, when translated into amino acid sequences, were only 10–20% divergent from CoV-1 and are likely to have come from a second strain of CoV-1 that also infects common green ti.

The discovery of four related closterovirus species co-infecting the same host which share a similar genome organization and perhaps similar biological properties presents a situation reminiscent to the GLRaV-4 group. Since these four CoVs were discovered through the intense study of a single ti plant, it is also plausible that additional CoV species exist. Some members of the GLRaV-4 group, however, were found to be serologically related (Ghanem-Sabanadzovic et al., 2012). There are currently no antisera raised against any of the CoVs that would allow experiments to be conducted to determine their serological relationships. Given the amount of sequence divergence between the currently known CoVs, particularly in their respective structural proteins, it seems unlikely that they will be serologically related.

Within the family *Closteroviridae*, LChV-1, GLRaV-7, and CoV-1 form a distinct monophyletic clade for which the genus “*Velarivirus*” has been proposed (Al Rwahnih et al., 2012; Martelli et al., 2012). Phylogenetic analyses placed CoV-2, CoV-3, and CoV-4 within this clade. We therefore propose that CoV-2, CoV-3, and CoV-4 also be members of the proposed genus “*Velarivirus*,” should it be ratified by the ICTV.

## Conflict of Interest Statement

The authors declare that the research was conducted in the absence of any commercial or financial relationships that could be construed as a potential conflict of interest.
